# “Antibiotics Are Not Automatic Anymore”—The French National Campaign To Cut Antibiotic Overuse

**DOI:** 10.1371/journal.pmed.1000080

**Published:** 2009-06-02

**Authors:** Benedikt Huttner, Stephan Harbarth

**Affiliations:** Infection Control Program, University of Geneva Hospitals and Medical School, Geneva, Switzerland

## Abstract

Benedikt Huttner and Stephan Harbarth discuss the implications of a new study that examined the impact of a national campaign in France to reduce antibiotic overuse.

**Linked Research Article**

This Perspective discusses the following new study published in *PLoS Medicine*:

Sabuncu E, David J, Bernède-Bauduin C, Pépin S, Leroy M, Boëlle P-Y, Watier L, Guillemot D (2009) Significant reduction of antibiotic use in the community after a nationwide campaign in France, 2002–2007. PLoS Med 6(6): e1000084. doi:10.1371/journal.pmed.1000084

Didier Guillemot and colleagues describe the evaluation of a nationwide programme in France aimed at decreasing unnecessary outpatient prescriptions for antibiotics. The campaign was successful, particularly in reducing prescriptions for children.

## Outpatient Antibiotic Use and Antibiotic Resistance

Antibiotic resistance is an important public health concern [1]. Antibiotics are one of the most commonly prescribed drug classes worldwide, with considerable variation in outpatient antibiotic use between countries [2]. Viral respiratory tract infections drive antibiotic overprescribing in the outpatient setting, and this overprescribing is also influenced by patient demand and expectations [3],[4].

There has been great interest in the public health community in avoiding unnecessary prescriptions, not only by providing treatment guidelines and decision support to physicians, but also by educating the public about appropriate antibiotic use. But the most effective strategy to achieve this goal remains unknown [5]. In the 1990s, one of the first national campaigns to reduce antibiotic prescribing resulted in a decrease in antibiotic use and penicillin-resistant *Streptococcus pneumoniae* in Iceland [6]. Since that time, there have been many other public campaigns, but published data about their impact remain scarce. Until now, the best evidence correlating a national campaign to a reduction in antibiotic use came from Belgium, where yearly mass media campaigns were associated with a 36% reduction in antibiotic prescriptions between 1999–2000 and 2006–2007 [7].

## The French National Campaign To Reduce Unnecessary Antibiotic Use

In a new study published in this issue of *PLoS Medicine*, Didier Guillemot and colleagues analyze the impact of a similar campaign in France, which used to be known for the highest rates of antibiotic use and pneumococcal resistance in Europe [8],[9]. In 2001, French policy makers and public health authorities launched a coordinated and multifaceted strategy for the control of antimicrobial resistance. One of the key interventions was a yearly campaign targeting the public via mass media, conveying the message that “Antibiotics Are Not Automatic” (especially for viral respiratory tract infections). Simultaneously, the streptococcal rapid antigen test and treatment guidelines were promoted among health care professionals. A substantial number of primary care physicians were targeted by one-on-one educational sessions known as “academic detailing”.

Analyzing prescribing data provided by the national health insurance (covering over 90% of the population) for two winters before and five winters after the launch of the first campaign, Guillemot and colleagues observed a decline by 26.5% in the number of antibiotic prescriptions, surpassing even the national target of a 25% reduction over five years. The decrease was seen in all French regions and age groups, with the highest decrease observed in children. Despite this decline in antibiotic use, a relative increase in fluoroquinolone use by 12.8% was observed, indicating the possibility that the choice of antibiotic may have become less appropriate for certain treatment indications.

## Strengths and Limitations of the Study

This study provides the largest and most sophisticated analysis published thus far correlating a nationwide public campaign to decreased antibiotic use over an extended period of time. The researchers address many potential sources of bias and confounding [10].

Nevertheless, some issues warrant further comment. Although it is likely that the reduction in antibiotic prescribing was causally related to the campaign, the ecological before-and-after study design cannot provide definitive proof of causality. As mentioned by the authors, in a few European countries a decrease in antibiotic use was also seen *without* major campaigns. The paper does not provide any information about surrogate markers that might explain the campaign's success, such as the public's awareness of the campaign or changes in knowledge and attitudes among physicians and the public. A limitation inherent to any multifaceted, complex intervention is that no clue is provided to identify which of the numerous interventions were most effective and could be recommended to other countries. Finally, although the impact on antibiotic prescriptions is impressive, the effect on antimicrobial resistance is still unclear and difficult to separate from the effect of the conjugate pneumococcal vaccine [11],[12].

## Cost Issues

Guillemot and colleagues report that developing and conducting the campaign cost 500 million euros over six years, which seems extremely costly considering that the Belgian campaign cost only about 400,000 euros per year [7]. But without more detailed cost-benefit analyses, this cost information should be interpreted with caution. It is, however, noteworthy that the reduction in antibiotic costs in France outweighed the cost of the public campaign to reduce prescribing.

The amount of money spent by the pharmaceutical industry to promote antibiotics should also be considered when assessing cost issues. Unfortunately, we do not have access to these figures for Europe. However, in the United States in 1998, it was estimated that pharmaceutical companies spent about US$1.6 billion to promote antibiotics, an amount that vastly exceeds any budget allocated to antibiotic campaigns [13].

## Is the French Experience an Example for Other Countries To Follow?

Overall, the results of the French public campaign are very encouraging and confirm those of the Belgian campaign: in countries with high baseline antibiotic use, sustained and multifaceted campaigns using mass media as well as targeting physicians can decrease antibiotic use substantially. Nonetheless, given the variations in antibiotic usage patterns, sociocultural determinants, and health care systems, different approaches are warranted for different countries [3]. Future campaign managers are certainly well-advised to apply social marketing concepts to identify the optimal approach for their own country [14]. We should not forget, however, that some national campaigns (e.g., Greece, Spain, England, Australia) *failed* to show a major impact on antibiotic prescriptions, although few of these data have been published [15],[16]. On the other hand, Sweden succeeded in reducing antibiotic use by a comprehensive program without a public campaign component [17].

## Next Steps

Since the ultimate goal of any campaign to reduce antibiotic use is to curb antibiotic resistance, more longitudinal and modeling studies are needed. Realistically, these studies may require individual patient data from small-scale surveillance networks, including monitoring of potential adverse effects of reduced prescribing.

All in all, the French have to be congratulated for their success story demonstrating that, if policy makers and opinion leaders are serious about reducing antibiotic misuse, this goal can be achieved by well-coordinated national efforts. Throughout the world, we should no longer accept antibiotic misuse and resistance as “automatic”.
